# Association Between Proton‐Pump Inhibitors and Muscle Strength in Older Adults: A Population‐Based Analysis

**DOI:** 10.1111/1751-2980.70068

**Published:** 2026-09-01

**Authors:** Jaakko Ollila, Konsta Teppo, Kimmo Salminen, Adriana Lääti, Jenni Vire, Matti Viitanen, Ville Langén

**Affiliations:** ^1^ Abdominal Center – Department of Gastroenterology Turku University Hospital and University of Turku Turku Finland; ^2^ Department of Internal Medicine University of Turku Turku Finland; ^3^ Heart Unit Satakunta Central Hospital Pori Finland; ^4^ Division of Medicine Turku University Hospital and University of Turku Turku Finland; ^5^ Department of Geriatric Medicine Turku University Hospital and University of Turku Turku Finland; ^6^ Department of Neurobiology, Care sciences and Society Karolinska Institutet Huddinge Sweden; ^7^ Theme Inflammation and Aging Karolinska University Hospital Stockholm Sweden

**Keywords:** muscle strength, older adults, physical function, proton‐pump inhibitors, sarcopenia

## Abstract

**Objective:**

We aimed to assess the association of proton‐pump inhibitor (PPI) use with muscle strength in older adults.

**Methods:**

All 1940‐born residents of Turku, Finland were surveyed during 2016 and 2017 as part of the New Turku Older Adults Study. Associations between PPI use, regardless of dosing pattern, and physical function were analyzed using Welch's *t*‐test, chi‐square test, and linear and logistic regression models. Models were initially run as crude, then adjusted for age, sex, and body mass index (Multivariable Model 1), and further adjusted for the Charlson Comorbidity Index (CCI) (Multivariable Model 2).

**Results:**

Among 616 participants (63.1% females), PPI users had lower mean handgrip strength (27.6 kg vs. 30.1 kg, *p* = 0.019), slower median 4‐m walking performance (3.7 s vs. 3.5 s, *p* = 0.017), and lower success in the armchair push–pull test (86.9% vs. 95.4%, *p* = 0.002) compared with non‐users. Crude regression analyses showed poorer performance across all outcomes among PPI users (all *p* < 0.05); however, this persisted only for the armchair push–pull test through Multivariable Model 2 (odds ratio 0.40, 95% confidence interval 0.19–0.85, *p* = 0.02). In the sensitivity analysis, replacing the CCI with non‐vitamin medication burden attenuated this association as well (*p* > 0.05).

**Conclusions:**

PPI use in older adults is significantly associated with lower muscle strength and poorer physical performance in less adjusted models, with these associations attenuating and disappearing with stepwise adjustment. These findings underscore the need to consider residual confounding in studies linking PPI use to adverse outcomes.

## Graphical abstract

In this population‐based study of 616 older adults, we analyzed the associations of proton‐pump inhibitor (PPI) use with handgrip strength, 4‐m walking performance, and armchair push–pull performance. After adjusted for age, sex, body mass index, and comorbidity burden using the Charlson Comorbidity Index (CCI), only the association with armchair push–pull performance remained statistically significant. However, when non‐vitamin medication burden replaced the CCI in the sensitivity analysis, even this association was attenuated and no longer statistically significant.

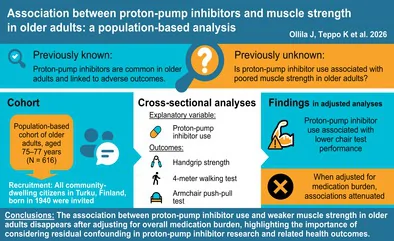

## 1 Introduction

More than three decades since their introduction into clinical practice, proton‐pump inhibitors (PPIs) have been the cornerstone of not only gastroesophageal reflux treatment but also gastroprotection in various clinical settings [1]. However, in recent years, there has been a growing shift toward a more conservative approach in their application [2]. Such change follows a series of concerning findings that link PPI use to increased risks of adverse outcomes, including pneumonia [3], osteoporosis [4], dementia [5], and all‐cause mortality [6]. Despite extensive research, many questions remain unanswered, particularly regarding the precise mechanisms behind these associations and the potential role of selection bias and residual confounding factors in these studies [7, 8]. Nonetheless, the accumulating evidence linking PPI use to negative health outcomes speaks against their indiscriminate long‐term use.

In light of ongoing discussions regarding potential adverse outcomes associated with PPI use, we hypothesized a possible association between PPI use and impaired muscle strength. This research question is particularly important for older adults, among whom gastroesophageal reflux is common, with a prevalence of 17.3% in individuals aged ≥ 50 years in a pooled meta‐analysis [9], and who may have limited awareness of the potential adverse effects associated with long‐term PPI use [10]. In this vulnerable population, reduced muscle strength is closely linked to frailty and diminished functional capacity, increasing the risk of falls that may lead to injury, fear of falling, loss of independence, and even death [11].

To our knowledge, no unselected population‐based studies have directly examined the association between PPI use and muscle strength [12]. To address this knowledge gap, we aimed to assess the relationship between PPI use and muscle strength in a population‐based cohort of 1940‐born older adults, who were identified from the local population registry in Turku, Southwest Finland, and invited to participate in our study at the age of 75–77 years.

## 2 Participants and Methods

### 2.1 Enrollment of the Participants

In 2010, the New Turku Older Adults Study (UTUVA) invited all 1940‐born, approximately 70‐year‐old residents of Turku, Southwest Finland to participate in a prospective cohort study (*N* = 1344). After excluding refusals, institutionalized individuals, and those with incomplete questionnaires, 956 (71.1%) participants were included in the initial study, which was conducted between 2011 and 2012. In the first follow‐up study that was conducted 5 years later, 707 participants underwent physical examination. This follow‐up introduced a set of variables related to muscle strength, which led to the selection of this time point for the data used in the present paper. Participants lacking data for the 4‐m walking test, at least one handgrip reading, the armchair push‐pull test, or other covariates used in our multivariable models were excluded. Therefore, 616 participants were included in the analyses presented in this paper. The flowchart of participant screening and enrollment is provided in Figure S1.

### 2.2 Health Interview and Examination

The study population underwent an examination protocol similar to that described in previous studies of this survey, which included postal questionnaires, interviews, and clinical examinations [13, 14, 15]. The examinations were conducted at the research center, where the 4‐m walking test, armchair push–pull test, and hand grip measurements were performed by trained research physicians.

Handgrip strength was measured using a hydraulic hand dynamometer (model J00105; Jamar, Patterson Medical, MN, USA). The participants were seated with their back straight, feet supported, and the elbow flexed at 90°, with the arm close to the body. Measurements were performed using the dominant hand. Two separate trials were conducted with a 30‐s rest interval, and both values were recorded. The mean value of the two measurements was used; if only one measurement was available, that value was used.

The 4‐m walking test was performed over a marked distance. Participants were instructed to walk at their usual pace, starting from a standing position behind the starting line. Timing began with the first foot movement and stopped when either foot crossed the finish line. The use of walking aids was permitted if necessary and was recorded.

The armchair push–pull test involved a 26‐kg armchair placed on a carpeted surface. The participant stood firmly behind the chair and was instructed to push it forward until the arms were fully extended, then pull it back to the starting position. Success was recorded if the participant was able to complete the task, even with difficulties.

Medications were documented using information obtained from the questionnaire, individual interviews, and prescriptions for each participant. These medications were then classified according to the specific Anatomical Therapeutic Chemical (ATC) classification system codes [16]. PPI use was defined as the use of any medication with an ATC code beginning with A02BC. Regular and as‐needed PPI use were distinguished for the descriptive data presented in Supplementary Table S1 but were not distinguished in the main analyses.

### 2.3 Statistical Analysis

All the statistical analyses were performed using Python 3.9.6 (Python Research Foundation, Wilmington, DE, USA), with the following packages: Pandas for data handling [17], *tableone* package for generating descriptive statistics [18], Matplotlib [19] and Plotnine [20] for data visualization, NumPy and SciPy for comparative analyses [21, 22], and Statsmodels for regression analysis [23]. A Jupyter notebook, running in Visual Studio Code (Microsoft, Redmond, WA, USA), was used as the integrated development environment. The ATC code validity and total drug counts were calculated separately in R 4.4.1 (R Core Team, Vienna, Austria) using custom functions for ATC validation and drug flagging. Vitamins (ATC code A11) were excluded from the medication burden definition and were not counted as drugs in the analyses. Furthermore, for participants using a PPI, the PPI itself was excluded from the non‐vitamin medication count to ensure that medication counts were comparable between PPI users and non‐users and reflected concomitant medications rather than the exposure of interest.

Continuous variables were assessed for normality by visual inspection of histograms. Normally distributed continuous variables were compared between PPI users and non‐users using Welch's *t*‐test, an adaptation of Student's *t*‐test that does not assume equal variances. Continuous variables wth a skewed distribution were compared using the Mann–Whitney *U*‐test. Categorical variables were presented as numbers and percentages or frequencies and were compared using the chi‐square test. Among all tests, handgrip strength was defined as the mean value of the two measurements when both were available. To further explore the associations between PPI use and physical function, we conducted linear regression for continuous outcomes and logistic regression for the binary outcome of success in the armchair push–pull test. The models were initially run as crude, followed by adjustment for age, sex, and body mass index (BMI) for Multivariable Model 1, and then additionally adjusted for comorbidity burden for Multivariable Model 2, which was assessed using the Charlson Comorbidity Index (CCI) [24]. As a sensitivity analysis, we repeated the fully adjusted Multivariable Model 2 using non‐vitamin medication burden as an alternative measure of comorbidity in place of the CCI to assess the robustness of the findings.

### 2.4 Ethics

The approvals (with the corresponding record numbers) for the UTUVA study were granted by the Ethical Committee of the Hospital District of Southwest Finland for the baseline study on 16 February 2010 (ETMK: 2/180/2010) and the 5‐year follow‐up on 17 March 2015 (ETMK: 39/1801/2015). All participants provided written informed consent in accordance with the Declaration of Helsinki.

## 3 Results

Overall, 616 participants were included in the analysis. The characteristics of the entire study population, as well as the subgroups with and without PPI medication, are presented in Table 1. PPI users did not differ significantly from non‐users regarding their age, sex distribution, or BMI (all *p* > 0.05). The PPIs used by participants in the study cohort were pantoprazole (*n* = 56), esomeprazole (*n* = 31), lansoprazole (*n* = 6), and omeprazole (*n* = 6). None of the participants in the study cohort were receiving rabeprazole. Rabeprazole, together with the four PPIs listed above, constitutes the five PPI agents currently available on the Finnish market [25]. Pantoprazole and esomeprazole accounted for the majority of PPI use, and most PPIs were prescribed for acid‐related disorders rather than gastroprotection. Detailed characteristics of PPI use, including dosage and indications, are presented in Table S1.

**TABLE 1:** Characteristics of study participants by proton‐pump inhibitor (PPI) usage status.

Variables	Overall (*N* = 616)	No PPI (*n* = 517)	PPI user (*n* = 99)	*p* value
Age, years (mean ± SD)	76.2 ± 0.1	76.2 ± 0.1	76.2 ± 0.1	0.918
Female (*n*, %)	389 (63.1)	320 (61.9)	69 (69.7)	0.174
BMI, kg/m^2^ (mean ± SD)	27.5 ± 4.6	27.4 ± 4.4	28.3 ± 5.4	0.107
Handgrip strength, kg (mean ± SD)	29.7 ± 10.0	30.1 ± 10.0	27.6 ± 9.7	0.019
4‐m walking test, s (median [IQR])	3.5 (3.1–4.1)	3.5 (3.0–4.0)	3.7 (3.1–4.6)	0.017
Success in armchair push–pull test (*n*, %)	579 (94.0)	493 (95.4)	86 (86.9)	0.002
Charlson comorbidity index (median [IQR])	3.0 (2.0–4.0)	3.0 (2.0–4.0)	4.0 (3.0–4.0)	< 0.001
Number of non‐vitamin medications (median [IQR])	4.0 (3.0–7.0)	4.0 (2.0–6.0)	6.0 (4.0–9.0)	< 0.001

*Note:* Normally distributed continuous variables were compared using the Welch's *t*‐test, non‐normally distributed continuous variables were compared using the Mann–Whitney *U*‐test, and categorical variables were compared using the chi‐square test. For PPI users, the PPI itself was excluded from the non‐vitamin medication count to avoid counting the exposure medication among concomitant medications, thereby enabling direct comparison between the two groups.

Abbreviations: BMI, body mass index; IQR, interquartile range; SD, standard deviation.

Compared with non‐users, PPI users had lower mean handgrip strength (27.6 kg ± 9.7 kg vs. 30.1 kg ± 10.0 kg, *p* = 0.019) and performed worse (i.e., took longer) on the 4‐met walking test (median 3.7 s [IQR 3.1–4.6 s] vs. 3.5 s [IQR 3.0–4.0 s], *p* = 0.017) (Table 1, Figures 1 and 2). In addition, a lower proportion of PPI users succeeded in the armchair push–pull test compared to non‐users (86.9% vs. 95.4%, *p* = 0.002) (Table 1, Figure 3).

**FIGURE 1:**
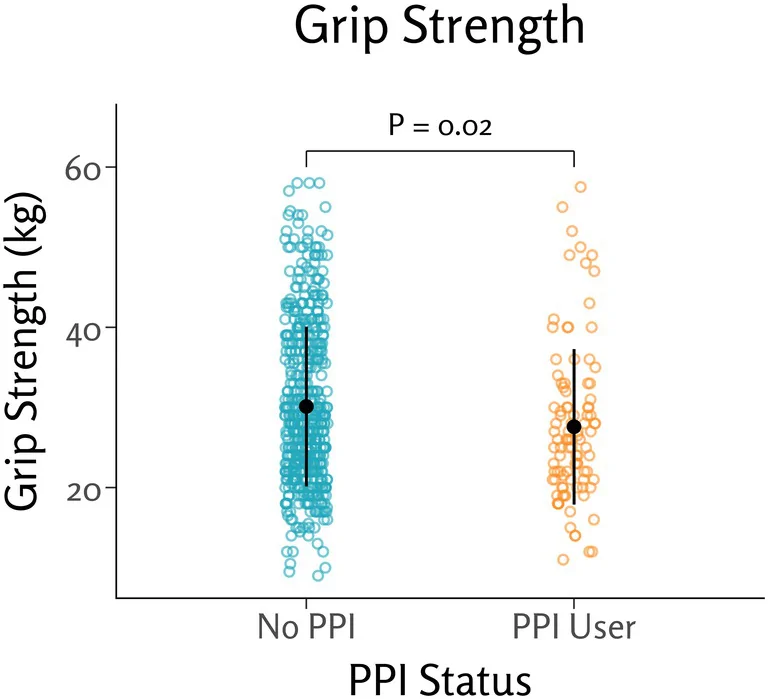
Handgrip strength in participants with and without proton‐pump inhibitor (PPI) use. Individual observations are shown as jittered points, with the group mean and standard deviation indicated.

**FIGURE 2:**
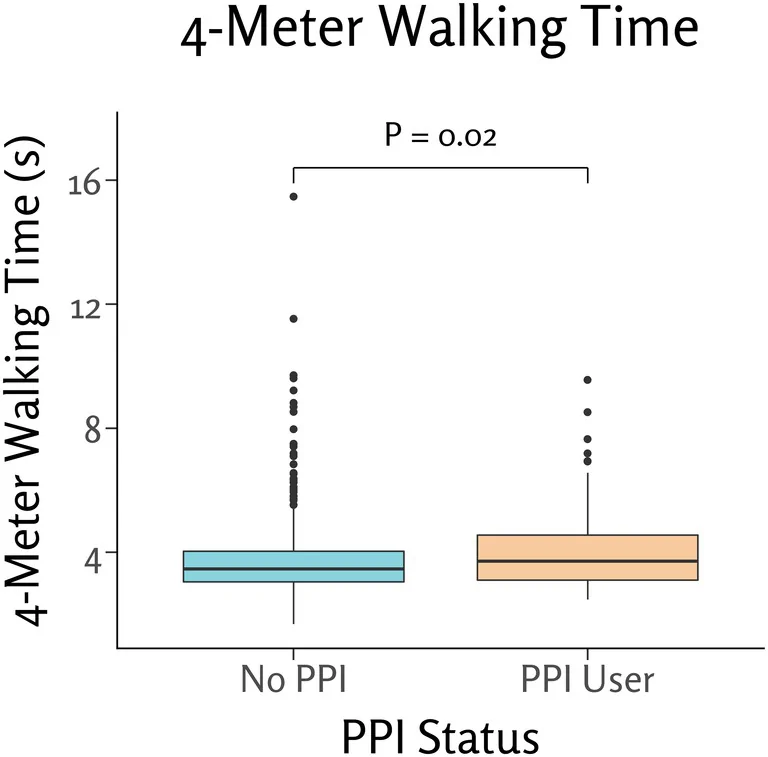
The 4‐meter walking test in participants with and without proton‐pump inhibitor (PPI) use. Box plots display the median and interquartile range.

**FIGURE 3:**
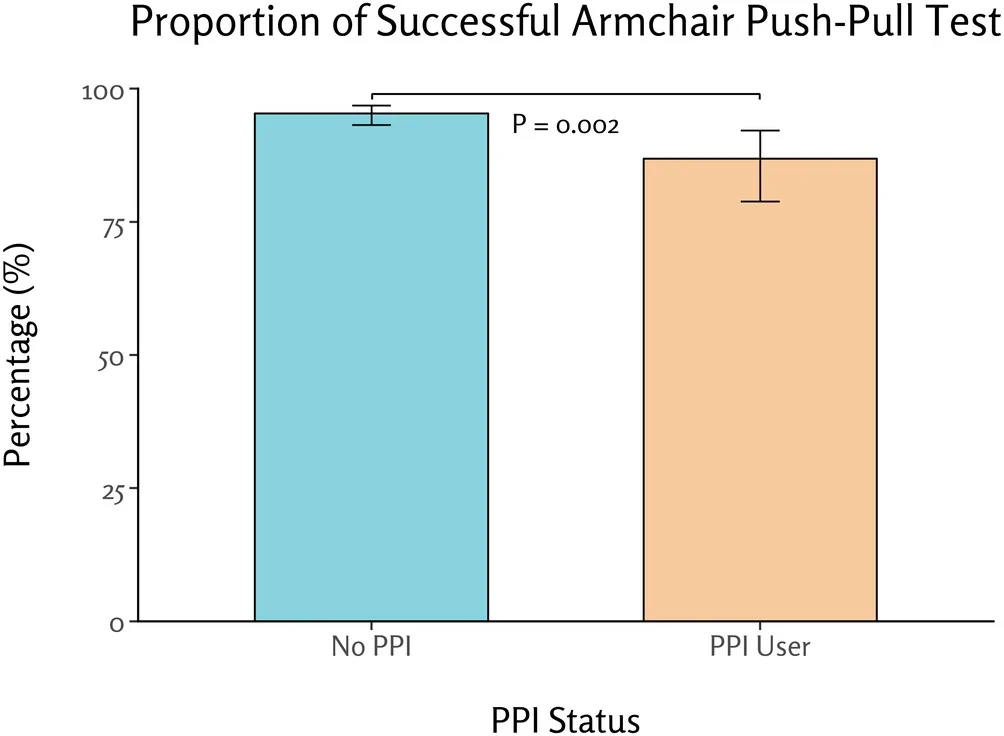
Proportion of participants successfully completed the armchair push–pull test according to proton‐pump inhibitor (PPI) use. Error bars represent 95% confidence intervals.

A crude regression model indicated that PPI use was significantly associated with lower handgrip strength (*β* = −2.53, *p* = 0.02), but the association was not statistically significant in the adjusted Multivariable Model 1 (*β* = −1.39, *p* = 0.06) (Table 2).

**TABLE 2:** Regression analyses of the association between proton‐pump inhibitor (PPI) usage and muscle strength in older adults.

Linear regression		Crude model			Multivariable Model 1			Multivariable Model 2		
Regressor	Outcome	*β*	SE	*p* value	*β*	SE	*p* value	*β*	SE	*p* value
PPI user (vs. no PPI)	Handgrip strength (kg)	−2.53	1.09	0.02	−1.39	0.72	0.06	−1.01	0.74	0.17
PPI user (vs. no PPI)	4‐m walking test (s)	0.33	0.14	0.02	0.26	0.13	0.06	0.14	0.14	0.32

Logistic regression		Crude model			Multivariable Model 1			Multivariable Model 2		
Regressor	Outcome	OR	95% CI	*p* value	OR	95% CI	*p* value	OR	95% CI	*p* value
PPI user (vs. no PPI)	Success in armchair push–pull test	0.32	0.16–0.66	0.002	0.37	0.18–0.76	0.01	0.40	0.19–0.85	0.02

*Note:* Multivariable Model 1 was adjusted for age, sex, and body mass index, and Multivariable Model 2 was additionally adjusted for the Charlson comorbidity index.

Abbreviations: CI, confidence interval; OR, odds ratio; SE, standard error.

In the 4‐m walking test, PPI use was associated with longer completion time in the crude model (*β* = 0.33, *p* = 0.02); however, this association was not statistically significant in the adjusted Multivariable Model 1 (*β* = 0.26, *p* = 0.06) (Table 2).

Moreover, PPI users had lower odds of succeeding in the armchair push–pull test in both the crude (odds ratio [OR] 0.32, 95% confidence interval [CI] 0.16–0.66, *p* = 0.002) and the adjusted Multivariable Model 1 (OR 0.37, 95% CI 0.18–0.76, *p* = 0.01) (Table 2). Notably, this association persisted to be statistically significant in the Multivariable Model 2 (OR 0.40, 95% CI 0.19–0.85, *p* = 0.02), which employed the CCI as the measure of comorbidity burden.

When the non‐vitamin medication burden was used as an alternative measure of comorbidity in the sensitivity analysis, the associations for handgrip strength, 4‐m walking speed, and success in the armchair push–pull test did not reach statistical significance (all *p* > 0.05) (Table S2).

## 4 Discussion

This cross‐sectional study investigated the association of PPI use with muscle strength in a population‐based cohort of individuals aged 75–77 years, all born in 1940. Physical performance was assessed using handgrip strength, the 4‐m walking test, and the armchair push–pull test. In unadjusted group comparisons, PPI users exhibited lower handgrip strength, slower walking performance, and a higher proportion of participants failing the armchair push–pull test compared to non‐users. In crude regression analyses, such differences were observed across all outcomes. With stepwise adjustment, the associations were attenuated, and only that with armchair push–pull performance persisted after adjustment for comorbidity burden using the CCI. When non‐vitamin medication burden was used as an alternative measure of comorbidity in sensitivity analyses, this association was also attenuated and did not remain statistically significant, suggesting that the observed differences might be explained by residual confounding rather than an independent association with PPI use.

Previous research on this topic is extremely limited. In a single‐center observational study of patients with heart failure, Ohori et al. found that PPI use was independently associated with muscle wasting, defined by a low appendicular skeletal muscle mass index, even after propensity score matching and multivariable adjustment in patients with heart failure. A clear limitation of that study is its highly selective sample, consisting solely of hospitalized heart failure patients admitted to a single tertiary care center, which limits generalizability and might have introduced selection bias [26]. Another study indirectly supporting a link between PPI use and sarcopenia was conducted by a research group investigating the biological mechanisms of sarcopenia using machine learning models. While not focused specifically on PPIs, the study identified PPI use as one of several predictive variables associated with sarcopenia in older adults. However, the relatively small sample size (*n* = 200) and the use of a complex machine learning model involving a large number of variables may have increased the risk of overfitting and limited the interpretability of the findings [27].

In our study, the population and methodology differed substantially from previous reports, as we assessed a well‐defined, population‐based cohort of 75–77 years with comprehensive information on medication use. Corroborating previous findings, the association between PPI use and armchair push–pull performance persisted after adjusted for comorbidity burden using the CCI in our study, although this association was attenuated when non‐vitamin medication burden was adjusted in sensitivity analyses. Reverse causality remains plausible, as individuals with poorer muscle strength or overall health may be more likely to use PPIs for conditions such as reflux, dyspepsia, or gastroprotection related to other medications. These findings could partially challenge some previous observational studies that reported an independent link between PPI use and adverse outcomes. Residual confounding, which has been discussed in several prior studies [7, 8], remains a key consideration, and future longitudinal studies are warranted to disentangle confounding factors and assess any independent long‐term effects of PPI use on muscle health.

Several limitations must be considered. First, handgrip strength and the armchair push–pull test, which serve as surrogate markers of broader muscle strength, could be influenced by a variety of conditions affecting upper limb function. Similarly, the 4‐m walking test is affected not only by muscle strength but also by other factors impacting gait, such as neurological or musculoskeletal disorders. Despite these limitations, we included all available measures in the analyses to provide a comprehensive assessment of physical function in the study population. Second, we acknowledge that medication burden that utilized in sensitivity analyses is not a validated comorbidity measure in the same sense as the CCI. Nevertheless, prescription history has been explored as a potential indicator of underlying morbidity in epidemiological research. Gedeborg et al. demonstrated in a large Swedish cohort that prior prescription use was common even among individuals without recorded CCI and was associated with increased mortality risk, suggesting that prescription history might capture health status beyond diagnosis‐based indices [28]. Third, the absolute number of PPI users was relatively small in this study (*n* = 99; 16.1%), which might have reduced precision in the regression analyses. Of note, the proportion of PPI users in the present study is broadly consistent with that reported previously [9]. Fourth, and most importantly, as this study was observational and cross‐sectional in design, causal inference is not possible, and prospective cohort studies or randomized controlled trials are needed to establish potential causal relationships.

In conclusion, our findings showed that associations between PPI use and lower muscle strength or physical performance were completely attenuated with incremental adjustment in the statistical models, suggesting that these differences might be explained by residual confounding rather than an independent association with PPI use. Further longitudinal studies are needed to establish whether the observed patterns in our unadjusted analyses could be explained by reverse causality or residual confounding. As the global population ages and PPI use remains widespread, such studies will be important for informing clinical practice. In the meantime, clinicians should rely on the current evidence base and clinical guidelines when considering deprescribing unnecessary medications, as part of best practice in the pharmacotherapy of older adults.

## Funding

Dr. Viitanen was supported by grants from the King Gustaf V's and Queen Victoria's Freemasons’ Foundation, the Perklén Foundation, and the State Research Funding of the Wellbeing Services County of Southwest Finland (grant no. 30045). Dr. Teppo was supported by grants from the Finnish Medical Foundation and the Finnish Foundation for Alcohol Studies. Dr. Langén was supported by grants from the State Research Funding of the Wellbeing Services County of Southwest Finland (grant no. 30055), the Uulo Arhio Memorial Foundation, and the Finnish Foundation for Cardiovascular Research. Dr. Lääti was supported by grants from the Uulo Arhio Memorial Foundation, the Urmas Pekkala Foundation, and the Betania Foundation. Dr. Ollila was supported by a grant from the Betania Foundation.

## Conflicts of Interest

Ville Langén has served as a speaker for Boehringer‐Ingelheim. Other authors declare no conflicts of interest.

## Supporting information


**Figure S1:** Flowchart of study population enrollment.

**Table S1:** Distribution of proton pump inhibitor (PPI) use in the study population.
**Table S2:** Regression analyses of the association between proton‐pump inhibitor (PPI) use and muscle strength in older adults.

## Data Availability

In the interest of research transparency and reproducibility, the Jupyter notebook used in this study, along with accompanying materials, has been made publicly available on GitHub and permanently archived on Zenodo under DOI: 10.5281/zenodo.15205659. These resources can be accessed via the DOI search portal at https://www.doi.org or directly through https://zenodo.org/records/21710372.
