# Supplementary material for: Association Between Proton‐Pump Inhibitors and Muscle Strength in Older Adults: A Population‐Based Analysis
Source: J Dig Dis. 2026 Sep 1;27(7-8):287–92. doi: 10.1111/1751-2980.70068 (PMC13716018; doi:10.1111/1751-2980.70068)
Supplement: Supplementary file 1 [file CDD-27-287-s002.docx]

**Table S1.** Distribution of proton pump inhibitor (PPI) use in the study population.

| PPIs | Total users (*n*) | Regular users (%) | Dose, mg (mean [range]) | Indications (%) | |
| --- | --- | --- | --- | --- | --- |
|  |  |  |  | Acid-related disorders | Gastroprotection |
| Esomeprazole | 31 | 77.4 | 29.6 (20–40) | 96.8 | 3.2 |
| Lansoprazole | 6 | 66.7 | 25 (15–30) | 100 | 0 |
| Omeprazole | 6 | 83.3 | 27.5 (20–40) | 100 | 0 |
| Pantoprazole | 56 | 75.0 | 35.6 (20–80) | 85.7 | 14.3 |

*Note:* Information on dose (mg) was missing among 18 users, and indication for use was not available from 12 users.

Table S2. Regression analyses of the association between proton-pump inhibitor (PPI) use and muscle strength in older adults.

| Linear regression | | Multivariable Model 2 | | |
| --- | --- | --- | --- | --- |
| Regressor | Outcome | *β* | SE | *p* value |
| PPI user (vs. no PPI) | Handgrip strength (kg) | −0.50 | 0.73 | 0.50 |
| PPI user (vs. no PPI) | 4-m walking speed test (s) | −0.002 | 0.13 | 0.99 |
| Logistic regression | | Multivariable Model 2 | | |
| Regressor | Outcome | OR | 95% CI | *p* value |
| PPI user (vs. no PPI) | Success in armchair push–pull test | 0.66 | 0.30–1.48 | 0.31 |

*Note:* This table presents a sensitivity analysis using non-vitamin medication burden in place of the Charlson Comorbidity Index as the measure of comorbidity. Only Multivariable Model 2 is shown, as the crude model and Multivariable Model 1 correspond to the main analyses. Multivariable Model 2 was adjusted for age, sex, body mass index, and the non-vitamin medication burden (PPIs excluded from the non-vitamin medication count).

Abbreviations: CI, confidence interval; OR, odds ratio.

Figure Legend

Figure S1. Flowchart of study population enrollment.
